# Environmental Lead Exposure and Attention Deficit/Hyperactivity Disorder Symptom Domains in a Community Sample of South Korean School-Age Children

**DOI:** 10.1289/ehp.1307420

**Published:** 2014-10-03

**Authors:** Soon-Beom Hong, Mee-Hyang Im, Jae-Won Kim, Eun-Jin Park, Min-Sup Shin, Boong-Nyun Kim, Hee-Jeong Yoo, In-Hee Cho, Soo-Young Bhang, Yun-Chul Hong, Soo-Churl Cho

**Affiliations:** 1 Division of Child and Adolescent Psychiatry, Department of Psychiatry, Seoul National University College of Medicine, Seoul, Republic of Korea; 2 Department of Psychiatry, Ilsan Paik Hospital, Inje University College of Medicine, Goyang, Republic of Korea; 3 Department of Psychiatry, Gil Medical Center, Gachon University of Medicine and Science, Incheon, Republic of Korea; 4 Department of Psychiatry, Gangnam Eulji Hospital, Eulji University, Seoul, Republic of Korea; 5 Department of Preventive Medicine, Seoul National University College of Medicine and Institute of Environmental Medicine, Seoul, Republic of Korea

## Abstract

**Background:**

Low-level environmental exposure to lead has been associated with both reduced intelligence and symptoms of attention deficit/hyperactivity disorder (ADHD). However, few studies have estimated the association of lead and intelligence independent of ADHD, and it is not clear from previous studies whether lead is associated with both inattention and impulsivity ADHD symptoms.

**Objectives:**

We estimated mutually adjusted associations of environmental lead exposure with both intelligence and ADHD symptoms, and associations between lead and specific ADHD-related domains.

**Methods:**

Blood lead concentrations were measured in a general population of 1,001 children 8–11 years of age. We used multivariable linear regression models to estimate associations of blood lead concentrations with IQ scores, teacher and parent ratings of ADHD symptoms, and measures of inattention and impulsivity. Models were adjusted for demographic variables and other environmental exposures (blood levels of mercury and manganese, urinary concentrations of cotinine, phthalate metabolites, and bisphenol A).

**Results:**

Associations of blood lead with lower IQ and higher impulsivity were robust to adjustment for a variety of covariates. When adjusted for demographic characteristics, other environmental exposures, and ADHD symptoms or IQ, a 10-fold increase in blood lead concentration was associated with lower Full-Scale IQ (–7.23; 95% CI: –13.39, –1.07) and higher parent- and teacher-rated hyperactivity/impulsivity scores (ADHD Rating Scale, 1.99; 95% CI: 0.17, 3.81 and 3.66; 95% CI: 1.18, 6.13, respectively) and commission errors (Continuous Performance Test, 12.27; 95% CI: –0.08, 24.62). Blood lead was not significantly associated with inattention in adjusted models.

**Conclusions:**

Low-level lead exposure was adversely associated with intelligence in school-age children independent of ADHD, and environmental lead exposure was selectively associated with impulsivity among the clinical features of ADHD.

**Citation:**

Hong SB, Im MH, Kim JW, Park EJ, Shin MS, Kim BN, Yoo HJ, Cho IH, Bhang SY, Hong YC, Cho SC. 2015. Environmental lead exposure and attention deficit/hyperactivity disorder symptom domains in a community sample of South Korean school-age children. Environ Health Perspect 123:271–276; http://dx.doi.org/10.1289/ehp.1307420

## Introduction

Lead is an environmental neurotoxicant known to interfere with brain development (Canfield et al. 2003; Lanphear et al. 2005; Surkan et al. 2007). Even low-level exposure to lead that is prevalent in daily living has been associated with reduced intelligence, impaired attention, and behavioral problems (Braun et al. 2006, 2008; Chen et al. 2007; Nevin 2007). Environmental lead exposure also has been associated with symptoms of attention deficit/hyperactivity disorder (ADHD) (Braun et al. 2006) and conduct disorder (Braun et al. 2008). However, given that intelligence itself may affect attention and behavior (Chen et al. 2007; Frazier et al. 2004), and that a negative influence of lead on intelligence has been well replicated (Needleman and Gatsonis 1990), at least part of the association between lead and attention and behavior may be mediated by its negative impact on intelligence. Previous studies examining the association between lead burden and neurobehavioral impairments have addressed this issue by adjusting for intelligence level as a covariate (Goodlad et al. 2013).

Thus far, the findings have been inconclusive on whether lead is associated with both the domains of ADHD symptoms (i.e., inattention and hyperactivity/impulsivity), or with only one of the two domains. Profound and pervasive neurological consequences of high-level lead exposure may result in an undifferentiated worsening of ADHD-like symptoms (Needleman 2009), but it has been suggested that low levels of lead exposure may have a greater influence on hyperactivity/impulsivity than on inattention (Nigg et al. 2008, 2010; Stewart et al. 2005, 2006). On the other hand, a recent meta-analysis examined the relation between lead burden and ADHD symptoms, and reported similar associations for the two symptom domains (Goodlad et al. 2013). Studies that aim to resolve this inconsistency require a sufficient sample size and measurements of both behavioral features, as well as neuropsychological indices that can sensitively distinguish between the domains of inattention and hyperactivity/impulsivity.

For example, the Continuous Performance Test (CPT) differentially measures sustained attention and response inhibition (Greenberg and Waldman 1993), and, compared with other neuropsychological tests, has been reported to be the most strongly correlated with a clinical diagnosis of ADHD (Frazier et al. 2004).

Thus, the primary aim of the current study was to differentiate the specific aspects of attentional and behavioral impairments associated with low-level lead exposure by measuring the intelligence level, ADHD-related behaviors (rated by multiple informants), and CPT performance in a large community sample of school-age children.

Secondarily, we aimed to confirm the link between environmental lead exposure and intelligence, independent of ADHD symptoms. If environmental lead exposure is associated with ADHD symptoms, given that ADHD symptoms may interfere with the child’s performance in intelligence test (Biederman et al. 2011), it would be reasonable to control for concurrent attention and behavioral problems when estimating the association between lead burden and intelligence, just as we need to control for intelligence level when examining the relation between lead burden and ADHD. Only a few studies investigating the association between lead burden and intelligence have achieved this by measuring both IQ and ADHD-related features in a large group of participants (e.g., Nigg et al. 2008).

Another important issue is potential confounding by other environmental exposures such as mercury, manganese, phthalate metabolites, and bisphenol A (BPA), as well as cotinine (a biomarker for tobacco exposure), each of which may be associated with ADHD (Bouchard et al. 2007; Braun et al. 2009; Cheuk and Wong 2006; Cho et al. 2010b, 2013; Hong et al. 2013; Julvez et al. 2010; Kim BN et al. 2009). Few studies have accounted for multiple environmental risk factors in the same population (Goodlad et al. 2013).

Using data from an initial subsample (*n* = 667) of the present study’s participants (*n* = 1,089), we previously explored the relationship between lead exposure and ADHD-related problems (Cho et al. 2010b). However, we did not have specific research questions about whether lead burden *a*) differentially affects intelligence and ADHD, *b*) predominantly affects impulsivity among the ADHD-related problems, and *c*) independently affects childhood neurobehavioral outcomes after adjusting for a wide range of potential confounders.

In a large community sample of school-age children, we investigated whether environmental lead exposure is associated with both reduced intelligence and ADHD symptoms, when adjusting for each other as covariates. Next, by adopting an established neuropsychological test of attention, we examined whether lead burden is associated predominantly with a specific domain of ADHD-related problems. Last, we adjusted for blood and urine concentrations of other environmental toxicants to examine potential confounding of associations between lead and cognitive and behavioral outcomes.

## Methods

*Study population*. The detailed protocol of the study was previously described elsewhere (Cho et al. 2013; Hong et al. 2013). Briefly, participants were recruited from five South Korean administrative regions (i.e., two urban cities, two industrial cities, and one rural district). We selected one to three schools that represented the local demographics, and sent letters of invitation to participate to parents of third- and fourth-graders. Before participation, parents and children were provided with detailed information about the study, and they provided written informed consent. The study protocol was approved by the institutional review board of the Seoul National University Hospital. The study was conducted in accordance with the Declaration of Helsinki.

*Measurement of cognitive and neurobehavioral function*. Each child was administered the following tests in a quiet room under the supervision of a licensed specialist in clinical psychology (M.S.S.) who was unaware of the child’s toxicant levels.

Children’s Verbal, Performance, and Full-Scale IQs were measured via the abbreviated form of the Korean Educational Development Institute’s Wechsler Intelligence Scales for Children (KEDI-WISC) (Park et al. 1996), which tests vocabulary, arithmetic, picture arrangement, and block design. The sum of the first two subtests’ age-adjusted scaled scores was used to estimate Verbal IQ, and the sum of the last two was used to estimate Performance IQ (Park et al. 1996). In the abbreviated battery, only the Full-Scale IQ is converted so that a score of 100 equates to the mean of the population. Scores from the abbreviated battery are known to strongly correlate with the WISC Full-Scale IQ, both in the original instrument (Kaufman 1976) and in the age-standardized Korean version (Kim and Kim 1986).

Children underwent a computerized CPT (Greenberg and Waldman 1993). In this test, the examinee is shown visual stimuli on a screen, one every 2 sec, for 100 msec. The examinee is required to respond to a square containing a triangle (target) but not to a square containing another square or a circle (nontarget). The target stimulus was presented 22.5% of the time during the first half of the test and 77.5% of the time during the second half of the test. The test assesses four major outcomes: *a*) omission errors (failure to respond to targets; a measure of inattention); *b*) commission errors (responding erroneously to nontargets; a measure of impulsivity); *c*) response time for correct responses; and *d*) the standard deviation of these response times (response time variability; a measure of consistency of attention). The CPT was standardized for age among Korean children and adolescents, and its reliability and validity have been established (Shin et al. 2000).

Parents and schoolteachers of the participating children completed the ADHD Rating Scale (ADHD-RS) (DuPaul et al. 1998) to evaluate symptoms of ADHD. The ADHD-RS contains 18 items adopted from the 18 symptoms listed in the *Diagnostic and Statistical Manual of Mental Disorders, Fourth Edition* (DSM-IV; American Psychiatric Association 2000) criteria for ADHD; accordingly, 9 items are related to inattention and 9 to hyperactivity and impulsivity, with each item rated from 0 to 3. Reliability and validity of the Korean version of the ADHD-RS have been well established among Korean children (So et al. 2002).

Parents also completed a questionnaire about demographic and other possibly relevant data, such as paternal education level and socioeconomic status.

*Measurement of blood lead*. Venous blood (5 mL) was collected from each child in metal-free tubes, and the samples were frozen and stored at –20°C. Before the analysis, the blood samples were brought to room temperature and vortexed well after thawing. The samples (0.1 mL) were diluted in 1.8 mL of matrix modifier reagent (composed of Triton X-100 and ammonium hydrogen phosphate dibasic), and then were mixed well using the vortex mixer and assayed using an atomic absorption spectrometer–graphite furnace (Analyst 800-Zeeman collection; PerkinElmer, Singapore). The limit of detection for lead using this method was 0.058 μg/dL. The samples were analyzed in duplicate, and we modeled the mean value of the two assays; when the coefficient of variation of the two assays was ≥ 10%, we analyzed the sample again (in duplicate) until we obtained a mean value with a coefficient of variation < 10%. The inter-run coefficients of variation were 1.74%.

Blood concentrations of mercury (Rhee et al. 2013) and manganese (Kim Y et al. 2009) as well as creatinine-standardized urinary concentrations of cotinine (Cho et al. 2010b, 2013), phthalate metabolites (mono-*n*-butyl phthalate, MnBP; mono-2-ethyl-5-oxohexyl phthalate, MEOHP; mono-2-ethylhexyl phthalate, MEHP) (Cho et al. 2010a; Kim BN et al. 2009) and BPA (Hong et al. 2013) were also measured and included as covariates in the analysis.

All analyses were carried out by Neodin Medical Institute (Seoul, Korea), a laboratory certified by the Korean Ministry of Health and Welfare. For the internal quality assurance and control, commercial reference materials were obtained from the German External Quality Assessment Scheme (G-EQUAS; Erlangen, Germany). As part of the external quality assurance and control, the institute passed the G-EQUAS operated by Friedrich-Alexander University; the Centers for Disease Control and Prevention (CDC)’s Lead and Multi-element Proficiency (LAMP) program (Atlanta, GA, USA); and the Quality Assurance Program operated by the Korea Occupational Safety and Health Agency (KOSHA, Korea).

*Statistical analyses*. Differences between the children included in and excluded from the main analyses were estimated using Student’s *t*-tests for continuous variables and chi-square tests for categorical variables. Blood lead concentrations (micrograms per deciliter) followed a log-normal distribution and were therefore log_10_-transformed for the statistical analysis. Simple and multiple linear regression analyses were performed to assess whether IQ (Verbal, Performance, and Full-Scale) or ADHD-related scores (ADHD-RS and CPT) were predicted by blood lead level. First, the analyses were unadjusted for potential confounders. Then, in the first adjusted model, the analyses were conducted while controlling for age (continuous), sex, residential region (urban, industrial, or rural), paternal education level (continuous), and socioeconomic status (yearly family income above or below US$25,000). Next, we added Full-Scale IQ (when predicting ADHD-RS or CPT scores) or ADHD-related variables (i.e., parent- and teacher-rated ADHD-RS inattention and hyperactivity/impulsivity scores, and CPT scores; when predicting IQ) as covariates. Last, we further controlled for log_10_-transformed concentrations of mercury, manganese, cotinine, phthalate metabolites, and BPA, in addition to the covariates described above. The confounding variables were selected based on known predictors of neurodevelopment and previous publications (Cho et al. 2013; Hong et al. 2013; Kim BN et al. 2009; Nigg et al. 2010). We performed complete case analyses to address missing data for model covariates. All statistical analyses were performed using SPSS 18.0 for Windows (SPSS Inc., IBM, Chicago, IL, USA). All results were considered to be statistically significant when *p*-value was < 0.05 (two-tailed).

## Results

A total of 1,089 children were initially recruited, among whom a blood sample was available for 1,005 children. Four children were further excluded from the analyses, because two had a history of seizure disorder, one of neonatal hypoxia, and one of head trauma accompanied by cerebral hemorrhage. The characteristics of the 1,001 participants are described in Table 1. The children excluded from the analysis were similar to the included population (data not shown), except for lower Full-Scale IQ (105.18 ± 12.47 compared with 110.07 ± 14.46, *p* = 0.002) and a higher proportion of rural residents (35.2% compared with 17.3%) and lower proportion of urban residents (33.0% compared with 43.4%; *p* < 0.001).

**Table 1:** Demographic characteristics of participants [mean ± SD or *n* (%) unless otherwise indicated] (total *n *= 1,001).

Characteristic	Value
Age (years)	9.05±0.70
Female	474 (47.4)
Region
Urban	434 (43.4)
Industrial	394 (39.4)
Rural	173 (17.3)
Paternal education
Years	13.75±2.20
Missing (*n*)	82
Yearly income
> $25,000	576 (62.1)
≤ $25,000	352 (37.9)
Missing (*n*)	73
IQ
Verbal	22.99±5.38
Performance	23.41±5.30
Full-Scale	110.07±14.46
Missing (*n*)	2
ADHD-RS, parent-rated
Inattention	5.31±4.91
Hyperactivity/impulsivity	3.52±3.96
Total	8.84±8.34
Missing (*n*)	66
ADHD-RS, teacher-rated
Inattention	5.08±6.53
Hyperactivity/impulsivity	3.61±5.46
Total	8.68±11.41
Missing (*n*)	59
CPT
Omission errors	61.13±25.00
Commission errors	71.57±28.23
Response time	46.72±12.67
Response time variability	68.04±35.10
Missing (*n*)	2
Environmental chemical concentrations [geometric mean(GSD)]^*a*^
Mercury (μg/L)	2.44(1.52)
Manganese (μg/L)	13.82(1.35)
Cotinine (μg/g Cr)	1.87(3.52)
MnBP (μg/g Cr)	51.66(1.85)
MEOHP + MEHP (μg/g Cr)	44.71(1.95)
BPA (μg/g Cr)	1.32(2.33)
Lead (μg/dL)	1.80(1.40)
Abbreviations: ADHD-RS, attention-deficit/hyperactivity disorder rating scale; CPT, Continuous Performance Test; Cr, creatinine; GSD, geometric standard deviation; MEHP, mono-2-ethylhexyl phthalate; MEOHP, mono-2-ethyl-5-oxohexyl phthalate; MnBP, mono-*n*-butyl phthalate. ^***a***^Numbers of missing observations for environmental chemicals: cotinine *n*=12, MnBP *n*=7, MEOHP + MEHP *n*=7, BPA *n*=7.	

A total of 839 children had complete data for all model covariates and outcomes (i.e., 25 variables shown in Table 1). Missing data ranged from 0% to 8.2%. Results from Little’s test (Little 1988) suggested that the data were missing completely at random (χ^2^ = 165.88, *p* = 0.876).

In this study, no measurement of blood lead was below the limit of detection. The minimum, 5th and 25th percentiles, median, 75th and 95th percentiles, and maximum of blood lead concentrations were 0.53, 1.03, 1.47, 1.81, 2.25, 3.01, and 6.16 μg/dL, respectively. Descriptive data for the distributions of the other environmental chemicals are presented in Supplemental Material, Table S1.

Without adjusting for any covariate, blood lead concentrations were negatively associated with Verbal, Performance, and Full-Scale IQ, and positively associated with both parent- and teacher-rated ADHD-RS inattention, hyperactivity/impulsivity, and total scores (Table 2, model 1). For the CPT, only commission errors and response time variability were significantly associated with blood lead concentrations.

**Table 2:** Associations between blood lead concentration and the scores from the KEDI-WISC intelligence test, ADHD-RS, and CPT.

Outcome	Model1^*a*^		Model2^*b*^		Model3^*c*^		Model4^*d*^		Model5^*e*^		Model6^*f*^	
	B (95% CI)	*p*-Value	B (95% CI)	*p*-Value	B (95% CI)	*p*-Value	B (95% CI)	*p*-Value	B (95% CI)	*p*-Value	B (95% CI)	*p*-Value
IQ
Verbal	–2.79 (–5.10, –0.47)	0.018	–2.64 (–4.99, –0.29)	0.028	–2.68 (–4.96, –0.40)	0.021	–2.64 (–4.98, –0.30)	0.027
Performance	–3.18 (–5.45, –0.90)	0.006	–2.59 (–4.96, –0.23)	0.032	–2.58 (–4.99, –0.18)	0.035	–2.04 (–4.51, 0.42)	0.104
Full-Scale	–8.34 (–14.54, –2.14)	0.008	–7.86 (–14.07, –1.65)	0.013	–7.84 (–13.84, –1.84)	0.010	–7.23 (–13.39, –1.07)	0.021
ADHD-RS, parent-rated
Inattention	3.09 (0.93, 5.26)	0.005	1.90 (–0.26, 4.06)	0.085					1.38 (–0.76, 3.53)	0.206	0.94 (–1.27, 3.16)	0.402
Hyperactivity/impulsivity	3.71 (1.98, 5.45)	<0.001	2.58 (0.82, 4.34)	0.004					2.40 (0.64, 4.17)	0.008	1.99 (0.17, 3.81)	0.032
Total	6.79 (3.12, 10.45)	< 0.001	4.46 (0.78, 8.13)	0.017					3.76 (0.09, 7.43)	0.044	2.90 (–0.86, 6.68)	0.131
ADHD-RS, teacher-rated
Inattention	6.15 (3.28, 9.02)	< 0.001	4.01 (1.16, 6.86)	0.006					2.87 (0.12, 5.61)	0.040	2.72 (–0.12, 5.56)	0.060
Hyperactivity/impulsivity	5.85 (3.46, 8.25)	< 0.001	4.09 (1.71, 6.47)	0.001					3.74 (1.36, 6.13)	0.002	3.66 (1.18, 6.13)	0.004
Total	12.01 (7.00, 17.02)	< 0.001	8.10 (3.18, 13.03)	0.001					6.61 (1.78, 11.45)	0.007	6.38 (1.36, 11.40)	0.013
CPT
Omission errors	9.42 (–1.31, 20.16)	0.085	5.14 (–6.02, 16.30)	0.366					1.96 (–8.95, 12.88)	0.724	0.68 (–10.53, 11.90)	0.905
Commission errors	21.09 (9.01, 33.16)	0.001	16.97 (4.73, 29.20)	0.007					13.86 (1.82, 25.89)	0.024	12.27 (–0.08, 24.62)	0.052
Response time	–2.86 (–8.31, 2.58)	0.303	–3.66 (–9.48, 2.15)	0.217					–4.31 (–10.13, 1.51)	0.146	–4.65 (–10.66, 1.34)	0.128
Response time variability	23.47 (8.44, 38.49)	0.002	14.14 (–0.75, 29.03)	0.063					11.09 (–3.66, 25.84)	0.140	9.13 (–5.96, 24.24)	0.235
Abbreviations: ADHD-RS, attention-deficit/hyperactivity disorder rating scale; B, unstandardized regression coefficient; CPT, Continuous Performance Test. ^***a***^Model1: unadjusted for covariates (*n*=1,001). ^***b***^Model2: adjusted for demographic variables (age, sex, residential region, paternal education level, and yearly income) (*n*=908). ^***c***^Model3: adjusted for demographic variables plus ADHD-RS scores (parent- and teacher-rated scores for inattention and hyperactivity/impulsivity) and CPT scores (*n*=851). ^***d***^Model4: adjusted for demographic variables and ADHD-RS and CPT scores, plus log_10_-transformed environmental chemical concentrations [blood mercury and manganese concentrations, and creatinine-standardized urine concentrations of cotinine, phthalate metabolites (MnBP, MEOHP + MEHP), and BPA] (*n*=839). ^***e***^Model5: adjusted for demographic variables and Full-Scale IQ (*n*=907). ^***f***^Model6: adjusted for demographic variables, Full-Scale IQ, and environmental chemical concentrations (*n*=895).												

Blood lead concentrations remained associated with lower verbal, performance, and Full-Scale IQ scores when adjusted for age, sex, residential region, paternal education level, and yearly income (Table 2, model 2), and the demographic variables plus parent- and teacher-rated ADHD-RS inattention and hyperactivity/impulsivity scores, and CPT scores (model 3). After additional adjustment for the other environmental chemicals (model 4), the association with Performance IQ was no longer statistically significant (–2.04; 95% CI: –4.51, 0.42), but Verbal and Full-Scale IQs were still significantly lower in association with a 10-fold increase in blood lead concentration (–2.64; 95% CI: –4.98, –0.30 and –7.23; 95% CI: –13.39, –1.07, respectively). In addition, the coefficients for the association between lead burden and IQs were similar throughout the analyses, suggesting that the association between environmental lead exposure and lower IQ is robust to confounding by other exposures, and that it is not mediated by effects on ADHD. No variance inflation factor was > 5.44.

When ADHD-RS scores were the outcome variables, however, adjusting for demographic information (Table 2, model 2) and Full-Scale IQ (model 5) attenuated the associations between blood lead concentrations and ADHD symptoms compared with the unadjusted model (model 1), particularly for inattention versus hyperactivity/impulsivity. After additional adjustment for other environmental chemicals (model 6), blood lead concentrations were still significantly associated with parent and teacher ratings for hyperactivity/impulsivity, but were no longer significant for inattention scores. When CPT scores were the outcome variables, blood lead concentrations were associated with the measure of impulsivity (i.e., commission errors) but not inattention (i.e., omission errors) when adjusted for demographic information (Table 2, model 2) and additionally for IQ (model 5). Although subsequent adjustment for blood or urine concentrations of other environmental chemicals attenuated the association (from 13.86; 95% CI: 1.82, 25.89 based on model 5 to 12.27; 95% CI: –0.08, 24.62 based on model 6), the findings still support a potential selective effect of environmental lead exposure on the impulsivity domain (vs. the inattention domain) of ADHD. No variance inflation factor was > 1.61.

To confirm the robustness of our findings, we tested a range of different combinations of covariates. First, we adjusted associations between lead and IQ only for the ADHD-RS hyperactivity/impulsivity scores and CPT commission errors among the ADHD-related variables (see Supplemental Material, Table S2, models 1 and 2). Next, we performed the analysis adjusting for each ADHD-RS score separately (i.e., parent-rated inattention, parent-rated hyperactivity, teacher-rated inattention, and teacher-rated hyperactivity) (see Supplemental Material, Table S3, models 1, 2, 3, and 4, respectively). Last, we estimated the association between lead burden and ADHD-related problems (parent and teacher ratings, CPT scores) adjusting for either Verbal IQ or Performance IQ, instead of the Full-Scale IQ (see Supplemental Material, Table S4). Across the various tests, the findings were similar, and did not alter the conclusion of the study.

## Discussion

The present study confirmed previous findings that low-level environmental lead exposure is associated with lower intelligence in school-age children. In addition, our results suggest that environmental lead exposure may be selectively associated with impulsivity among the clinical features of ADHD.

The negative associations between lead burden and IQ were robust, as evidenced by the largely comparable strength and statistical significance of the associations with and without the covariates. Most studies investigating the impact of lead on intelligence have relied on observational design rather than a randomized trial, which limits our ability to infer causal relationships. In this regard, it is noteworthy that concurrent rather than earlier blood lead levels have shown the strongest association with IQ in school-age children (Chen et al. 2005, 2007; Lanphear et al. 2005). Generally speaking, exposure to neurotoxicants earlier in life has a more critical effect on the brain; this is also the case for many other environmental chemicals (Braun et al. 2006, 2011; Julvez et al. 2010). Therefore, regarding the reported correlations between concurrent lead exposure and child IQ, an alternative hypothesis may need to be taken into account when inferring the direction of causality. For example, a child who is experiencing symptoms of ADHD is perhaps at a higher chance of undergoing greater amounts of environmental lead exposure (Goodlad et al. 2013). In addition, ADHD symptoms may also negatively affect the child’s performance in intelligence tests (Frazier et al. 2004). Therefore, according to this hypothetical scenario, a child’s inattentive, hyperactive, or impulsive symptoms would be responsible for both the high level of lead exposure and low intelligence. The best way to test for the direction of causality would be through a prospective randomized trial of different levels of lead exposure, which is not realistic for ethical reasons (Goodlad et al. 2013). Alternatively, we controlled for ADHD-related problems when examining the relation between lead burden and intelligence, just as we controlled for intelligence when testing the relation between lead burden and behavior. A clear determination of causal direction or the potential influence of mediation may not be possible based on our analysis, but our findings suggest that environmental lead exposure has independent effects on both intelligence and ADHD. In addition, as mentioned above, lead exposure is one of the few for which concurrent rather than earlier exposure has shown the strongest association with childhood cognition and behavior. Therefore, studies investigating associations between neurobehavioral outcomes in children and other environmental risk factors should consider potential confounding by environmental exposure to lead.

Another important issue that remains equivocal in the literature is the specific domain of neurobehavioral impairment associated with lead exposure. Previous findings have been inconsistent regarding whether lead contributes to inattention in addition to impulsivity (Goodlad et al. 2013; Nigg et al. 2008, 2010; Stewart et al. 2005, 2006). In the present study, blood lead concentrations were significantly associated with all ADHD-RS domains before adjustment, but associations with inattention scores were notably attenuated after adjustment for a range of covariates. Chen et al. (2007) reported evidence consistent with a direct effect of blood lead concentrations on concurrent externalizing problems at 7 years of age that did not appear to be mediated through an effect of lead on IQ (Chen et al. 2007). The Oswego Children’s Study also addressed the issue of impulsivity versus inattention in a study of postnatal lead exposure as well as prenatal polychlorinated biphenyl (PCB) exposure, and reported that both exposures were associated predominantly with impulsivity, as measured by the CPT (Stewart et al. 2005) and the Differential Reinforcement of Low Rates task (DRL) (Stewart et al. 2006). Our findings support and extend those of some previous reports (Chen et al. 2007; Nigg et al. 2008, 2010; Stewart et al. 2005, 2006) by showing that low-level lead exposure was associated with impulsivity, but not inattention, in school-age children, and that the association persisted when adjusted for IQ and other environmental exposures that may mediate or confound neurobehavioral effects of lead.

As predicted, associations with lead exposure varied between the specific domains of ADHD as measured by the CPT. Specifically, blood lead levels were selectively associated with commission errors, a measure of impulse control. In addition, decreased CPT response time in combination with increased commission errors is considered to indicate that the child is highly impulsive (Butler and Montgomery 2005). Indeed, our results indicated that the direction of association between blood lead level and CPT response time was consistently negative, albeit not statistically significant, and these findings further support the robustness of the association between lead burden and impulsivity. Lead was also associated with behavioral measures of impulsivity (i.e., parent and teacher ratings on the ADHD-RS) after adjustment for potential confounders, consistent with higher commission scores on the CPT.

Reduction in lead burden has been linked to a decline in antisocial behavior (e.g., violence and crime) (Nevin 2000, 2007; Stretesky and Lynch 2001) but not to a decreased prevalence of ADHD (Goodlad et al. 2013). Several possible explanations can be drawn from the current findings. First, low-level exposure to lead may specifically affect impulsivity, rather than contributing to all ADHD domains. Second, previously reported associations between low-level lead exposure and ADHD may have been confounded by exposures to other neurotoxicants (Claus Henn et al. 2012; Kim Y et al. 2009) that have increased in prevalence as environmental lead exposures have declined. Third, it is worth noting that there are limited data on trends in ADHD. The first national study in the United States that relied on DSM-IV diagnostic criteria for ADHD was published only in 2007 (Froehlich et al. 2007); there are no data on trends using DSM-IV criteria.

The present study did have some limitations. First, the cross-sectional nature of the study design and a single, concurrent measurement of lead and other environmental chemicals limit our ability to conclude that exposures preceded the outcomes. Second, other environmental exposures that have been linked to ADHD in prior studies were not adjusted for, such as prenatal tobacco exposure (Nigg 2006) and organophosphate exposure (Bouchard et al. 2010). Prenatal tobacco exposure is an important potential confounder that also may interact with lead to modify the association between lead exposure and ADHD (Froehlich et al. 2009). A higher level of organophosphate pesticide exposure was suggested to contribute to the childhood burden of ADHD (Bouchard et al. 2010). In addition, failure to measure at least one PCB congener represents a weakness of the study given potential effects of PCBs on the outcomes of interest (Stewart et al. 2005, 2006). Third, we did not evaluate possible synergistic or interactive effects of other environmental exposures, which should be a focus of future studies. Fourth, other potential confounding variables may need to be controlled for, including birth complications or family characteristics.

In conclusion, we demonstrated that low-level lead exposure was associated with lower intelligence in school-age children independent of associations with attentional and behavioral problems, and that environmental lead exposure was selectively associated with impulsivity among the clinical features of ADHD. The findings further highlight the need for understanding the mental health effects of co-exposure to different combinations of environmental neurotoxicants.

## Supplemental Material

(366 KB) PDF
