# Supplementary material for: Environmental Lead Exposure and Attention Deficit/Hyperactivity Disorder Symptom Domains in a Community Sample of South Korean School-Age Children
Source: Environ Health Perspect. 2014 Oct 3;123(3):271–6. doi: 10.1289/ehp.1307420 (PMC4348739; doi:10.1289/ehp.1307420)
Supplement: (366 KB) PDF [file ehp.1307420.s001.508.pdf]

Environmental Health Perspectives doi:10.1289/ehp.1307420

Supplemental Material

Environmental Lead Exposure and Attention-Deficit/Hyperactivity Disorder Symptom Domains in a Community Sample of South Korean School-Age Children

Soon-Beom Hong, Mee-Hyang Im, Jae-Won Kim, Eun-Jin Park, Min-Sup Shin, Boong-Nyun Kim, Hee-Jeong Yoo, In-Hee Cho, Soo-Young Bhang, Yun-Chul Hong, and Soo-Churl Cho

Table S1. Biological levels of environmental chemicals.

Analyte	% > LOD[a]	Geometric mean ± GSD	Minimum	5th %ile	25th %ile	50th %ile	75th %ile	95th %ile	Maximum
Lead (μg/dL)	100% > 0.05	1.80 ± 1.40	0.53	1.03	1.47	1.81	2.25	3.01	6.16
Mercury (μg/L)	100% > 0.20	2.44 ± 1.52	0.62	1.19	1.84	2.47	3.20	4.85	8.59
Manganese (μg/L)	100% > 1.18	13.82 ± 1.35	4.25	8.15	11.45	14.13	17.23	21.45	31.50
Cotinine (μg/L)	60% > 1.00	1.83 ± 3.57	N/A	N/A	N/A	1.90	4.50	17.80	248.00
Cotinine (μg/g Cr)	N/A	1.87 ± 3.52	0.18	0.33	0.63	1.74	4.53	18.00	107.36
MnBP (μg/L)	100% > 0.44	50.80 ± 2.16	2.19	13.82	31.83	50.24	82.42	175.86	1298.29
MnBP (μg/g Cr)	N/A	51.66 ± 1.85	3.84	18.80	36.47	53.40	76.18	132.93	914.96
MEOHP (μg/L)	100% > 0.04	19.56 ± 2.42	0.10	3.95	12.09	20.77	34.94	75.07	346.62
MEOHP (μg/g Cr)	N/A	19.89 ± 2.07	0.09	5.93	13.09	20.80	30.79	60.70	498.23
MEHP (μg/L)	100% > 0.01	23.99 ± 2.30	0.50	5.94	14.80	25.38	41.69	82.23	448.31
MEHP (μg/g Cr)	N/A	24.39 ± 1.96	1.11	8.21	16.39	25.27	36.41	67.71	762.26
Bisphenol A (μg/L)	100% > 0.15	1.30 ± 2.45	0.16	0.33	0.67	1.23	2.29	5.65	125.16
Bisphenol A (μg/g Cr)	N/A	1.32 ± 2.33	0.14	0.36	0.75	1.28	2.19	5.35	300.15

Abbreviations: Cr, creatinine; GSD, geometric standard deviation; LOD, limit of detection; MEHP, mono-2-ethylhexyl phthalate; MEOHP, mono-2-ethyl-5-oxohexyl phthalate; MnBP, mono-n-butyl phthalate.

[a]The concentrations of all samples were higher than the LOD except for urinary cotinine levels. For concentrations below the LOD, half of the LOD value was used for statistical analyses.

Table S2. Adjusted associations between blood lead concentration and the scores from the intelligence test controlling for ADHD-RS hyperactivity/impulsivity scores and CPT commission errors.

Outcome	Model 1[a] B (95% CI)	*P*-value	Model 2[b] B (95% CI)	*P*-value
IQ				
Verbal	-2.48 (-4.77, -0.20)	0.033	-2.45 (-4.79, -0.10)	0.041
Performance	-2.37 (-4.79, 0.04)	0.054	-1.81 (-4.29, 0.65)	0.150
Full scale	-7.11 (-13.17, -1.05)	0.021	-6.47 (-12.69, -0.25)	0.041

Abbreviations: ADHD-RS, attention-deficit/hyperactivity disorder rating scale; B, unstandardized regression coefficient; CI, confidence interval; CPT, continuous performance test; IQ, intelligence quotient; SE, standard error.

[a]Model 1: adjusted for demographic variables (age, gender, residential region, paternal education level, and yearly income) plus ADHD-RS scores (parent- and teacher-rated scores for hyperactivity/impulsivity) and CPT commission errors (n = 851). [b]Model 2: adjusted for demographic variables and ADHD-RS hyperactivity/impulsivity scores and CPT commission errors, plus log10-transformed environmental chemical concentrations [blood mercury and manganese concentrations, and creatinine-standardized urine concentrations of cotinine, phthalate metabolites (MnBP, MEOHP + MEHP), and bisphenol A (n = 839).

Table S3. Adjusted Associations between blood lead concentration and the scores from the intelligence test, ADHD-RS and CPT controlling for each of the ADHD-RS scores and biological levels of selected environmental toxins associated with each outcome measure.

Outcome	Model 1[a] B (95% CI)	*P*-value	Model 2[b] B (95% CI)	*P*-value	Model 3[c] B (95% CI)	*P*-value	Model 4[d] B (95% CI)	*P*-value
IQ								
Verbal	-2.18 (-4.45, 0.08)	0.060	-2.26 (-4.57, 0.03)	0.054	-2.13 (-4.44, 0.18)	0.071	-2.58 (-4.95, -0.20)	0.033
Performance	-2.46 (-4.80, -0.12)	0.039	-2.48 (-4.84, -0.13)	0.038	-2.20 (-4.61, 0.20)	0.072	-2.48 (-4.92, -0.04)	0.046
Full scale	-6.97 (-12.93, -1.00)	0.022	-7.11 (-13.15, -1.07)	0.021	-6.38 (-12.46, -0.29)	0.040	-7.52 (-13.77, -1.27)	0.018

Abbreviations: ADHD-RS, attention-deficit/hyperactivity disorder rating scale; B, unstandardized regression coefficient; CI, confidence interval; CPT, continuous performance test; IQ, intelligence quotient; SE, standard error.

[a]Model 1: adjusted for demographic variables (age, gender, residential region, paternal education level, and yearly income) plus parent-rated ADHD-RS inattention score, and CPT scores (n = 902). [b]Model 2: adjusted for demographic variables plus parent-rated ADHD-RS hyperactivity/impulsivity score, and CPT scores (n = 902). [c]Model 3: adjusted for demographic variables plus teacher-rated ADHD-RS inattention score, and CPT scores (n = 855). [d]Model 4: adjusted for demographic variables plus teacher-rated ADHD-RS hyperactivity/impulsivity score, and CPT scores (n = 855).

Table S4. Adjusted associations between blood lead concentration and the scores from the ADHD-RS and CPT controlling for verbal or performance IQ.

**Outcome**	**Model 1[a] B (95% CI)**	***P*-value**	**Model 2[b] B (95% CI)**	***P*-value**
ADHD-RS, parent-rated				
Inattention	1.39 (-0.74, 3.53)	0.201	1.64 (-0.52, 3.81)	0.137
Hyperactivity/impulsivity	2.42 (0.66, 4.19)	0.007	2.50 (0.73, 4.26)	0.006
Total	3.79 (0.13, 7.45)	0.042	4.11 (0.43, 7.80)	0.029
ADHD-RS, teacher-rated				
Inattention	3.00 (0.25, 5.76)	0.032	3.36 (0.56, 6.16)	0.019
Hyperactivity/impulsivity	3.82 (1.43, 6.20)	0.002	3.88 (1.49, 6.26)	0.001
Total	6.82 (1.97, 11.67)	0.006	7.24 (2.35, 12.12)	0.004
CPT				
Omission errors	2.74 (-8.25, 13.73)	0.625	3.13 (-7.91, 14.18)	0.578
Commission errors	14.28 (2.24, 26.32)	0.020	15.28 (3.10, 27.45)	0.014
Response time	-4.02 (-9.85, 1.80)	0.176	-3.99 (-9.82, 1.83)	0.179
Response time variability	11.84 (-2.95, 26.63)	0.117	12.47 (-2.38, 27.33)	0.100

Abbreviations: ADHD-RS, attention-deficit/hyperactivity disorder rating scale; B, unstandardized regression coefficient; CI, confidence interval; CPT, continuous performance test; IQ, intelligence quotient; SE, standard error.

[a]Model 1: adjusted for demographic variables (age, gender, residential region, paternal education level, and yearly income) and verbal IQ (n = 907). [b]Model 2: adjusted for demographic variables and performance IQ (n = 907).
